# A new method for reconstructing brain morphology: applying the brain-neurocranial spatial relationship in an extant lungfish to a fossil endocast

**DOI:** 10.1098/rsos.160307

**Published:** 2016-07-20

**Authors:** Alice M. Clement, Robin Strand, Johan Nysjö, John A. Long, Per E. Ahlberg

**Affiliations:** 1 Department of Organismal Biology, Evolutionary Biology Centre, Uppsala University, Norbyvägen 18A, 752 36 Uppsala, Sweden; 2 Department of Sciences, Museum Victoria, GPO Box 666, Melbourne 3001, Victoria, Australia; 3 Centre for Image Analysis, Department of Information Technology, Uppsala University, Lägerhyddsvägen 2, 751 05 Uppsala, Sweden; 4 School of Biological Sciences, Flinders University, PO Box 2100, Adelaide 5001, South Australia, Australia

**Keywords:** endocast, brain, *Neoceratodus*, *Rhinodipterus*, microtomography, palaeoneurology

## Abstract

Lungfish first appeared in the geological record over 410 million years ago and are the closest living group of fish to the tetrapods. Palaeoneurological investigations into the group show that unlike numerous other fishes—but more similar to those in tetrapods—lungfish appear to have had a close fit between the brain and the cranial cavity that housed it. As such, researchers can use the endocast of fossil taxa (an internal cast of the cranial cavity) both as a source of morphological data but also to aid in developing functional and phylogenetic implications about the group. Using fossil endocast data from a three-dimensional-preserved Late Devonian lungfish from the Gogo Formation, *Rhinodipterus*, and the brain-neurocranial relationship in the extant Australian lungfish, *Neoceratodus*, we herein present the first virtually reconstructed brain of a fossil lungfish. Computed tomographic data and a newly developed ‘brain-warping’ method are used in conjunction with our own distance map software tool to both analyse and present the data. The brain reconstruction is adequate, but we envisage that its accuracy and wider application in other taxonomic groups will grow with increasing availability of tomographic datasets.

## 1. Introduction

The field of palaeoneurology was established close to a century ago, led by Tilly Edinger, who described a natural mould of the internal cranial cavity (termed an endocast) of *Nothosaurus*, a marine reptile from the Triassic Period [1]. At this same time, a remarkable fossilized hominid brain was described from South Africa [2]. While they do not usually contain the actual brain preserved inside them (except in some exceptional circumstances, e.g. [3]), fossil cranial endocasts have long been studied as a proxy for the brain, or indeed in their own right as a source of comparable morphological diversity between taxa. Previously, scientists often had to destroy their specimens to access the necessary information about their internal anatomy using time-consuming and destructive serial grinding methods [4]. Some notable early works on fishes include those by researchers from the ‘Stockholm School’ [5,6]. However, palaeoneurology has been undergoing something of a revival recently due to advances in, and increased accessibility to, modern technology. More specifically, ‘virtual palaeontology’ acquires three-dimensional information using tomographic scanning techniques (e.g. micro-, nano- and synchrotron) after which data processing and image segmenting can be performed using a variety of software packages.

**Figure 1.**
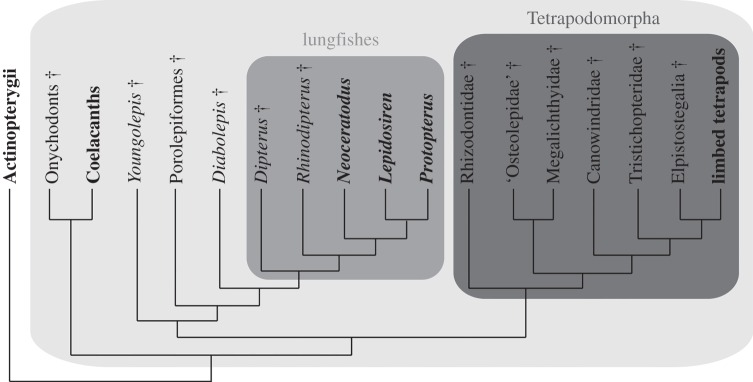
Composite tree showing relationships of major early sarcopterygian groups. Extant groups highlighted in bold, extinct groups indicated by a dagger (†), pale grey box indicates Sarcopterygii, mid grey indicates Dipnoi, darkest grey indicates Tetrapodomorpha. Phylogenetic relationships collated from [7–10].

Lungfish, or dipnoans as they are also known, are a group of fish that first appeared in the Devonian Period almost 400 Ma. They are osteichthyans (bony fishes) and belong to the sarcopterygian ‘lobe-fins’ alongside coelacanths and tetrapods (limbed vertebrates and their descendants). Lungfish have been confirmed as the closest living taxa to the tetrapods (figure 1) using both morphological and molecular evidence [11–13]. There are just six extant species in three genera, found in Africa (*Protopterus*), South America (*Lepidosiren*) and Australia (*Neoceratodus*). *Neoceratodus* is the sole remaining member of its family that is thought to have diverged from the Lepidosireidae over 250 Ma [14].

The cranial endocasts of a large range of fossil taxa are now available, ranging from those of the extinct armoured fishes known as ‘placoderms’ (e.g. *Kujdanowiaspis* [5], *Buchanosteus* [15]), to primitive sharks (*Cladodoides* [16], *Cobelodus,* [17]), various early actinopterygians [18–20] and sarcopterygian fishes and tetrapods [5,7,21,22]. However, of particular interest to the following study are the endocasts of fossil lungfishes; the first illustrated was that of *Chirodipterus wildungensis* from the Upper Devonian of Germany [23], then followed by those of the Early Devonian *Dipnorhynchus* [24,25], and partial endocast information from *Holodipterus, Chirodipterus* and *Griphognathus* from the Late Devonian Gogo Formation in north Western Australia [26,27]. Also hailing from the Gogo Formation, *Rhinodipterus kimberleyensis,* was the first virtual lungfish endocast described [28], followed closely by extensive work on *Dipterus* from the Middle Devonian of Scotland [29].

Generally, the brain-neurocranial relationships in vertebrate taxa such as mammals [30] or reptiles and birds [31,32] are thought to be ‘a better fit’ and more tightly packed in contrast to those in fishes. Indeed, the physical growth of the brain in amphibians and reptiles is ‘a factor in molding the skull’ and their endocasts closely ‘reproduce many brain features’ [32, pp. 368–369]. In fishes on the other hand, this idea has drawn further support from reports of some shark brains occupying as little as 6% of their cranial cavity [33], and the coelacanth *Latimeria* reported to occupy a mere 1% [34]. However, it has been noted that the endocast morphologies of some early actinopterygian fishes indicate a close match between certain regions of the brain [18,19], as was also noted for the dipnoan forebrain [5]. In fact, recent work by Clement *et al*. [35] shows that the brain-neurocranial relationship can differ drastically across fishes, with the brain of *Neoceratodus* the extant Australian lungfish shown to occupy more than 80% of its cranial cavity.

In the past, attempts to reconstruct the morphology of the brain from the endocast have been based simply on an intuitive ‘best fit’ of gnathostome brain anatomy to the shape of the cranial cavity (e.g. [6, fig. 87*b*]). The advent of tomographic data, which allow us to quantify the exact spatial and positional relationship between brain and endocast in extant taxa [35], creates new possibilities for making the reconstruction process on a more explicit and rigorous basis. Here, we use quantitative spatial data from the brain-neurocranial relationship in the extant Australian lungfish *Neoceratodus* [35] as a template for reconstructing the shape of the brain of the Devonian lungfish *Rhinodipterus* on the basis of a CT scan of its endocast [28]. We are able to do this by means of a newly developed registration and warping analysis detailed herein. This provides a test case for evaluating the utility of the technique in its simplest form, based on comparison with a single extant taxon; the potential for phylogenetically constrained multi-taxon comparisons is considered in the discussion.

## 2. Material and methods

### 2.1. Study material

**Figure 2.**
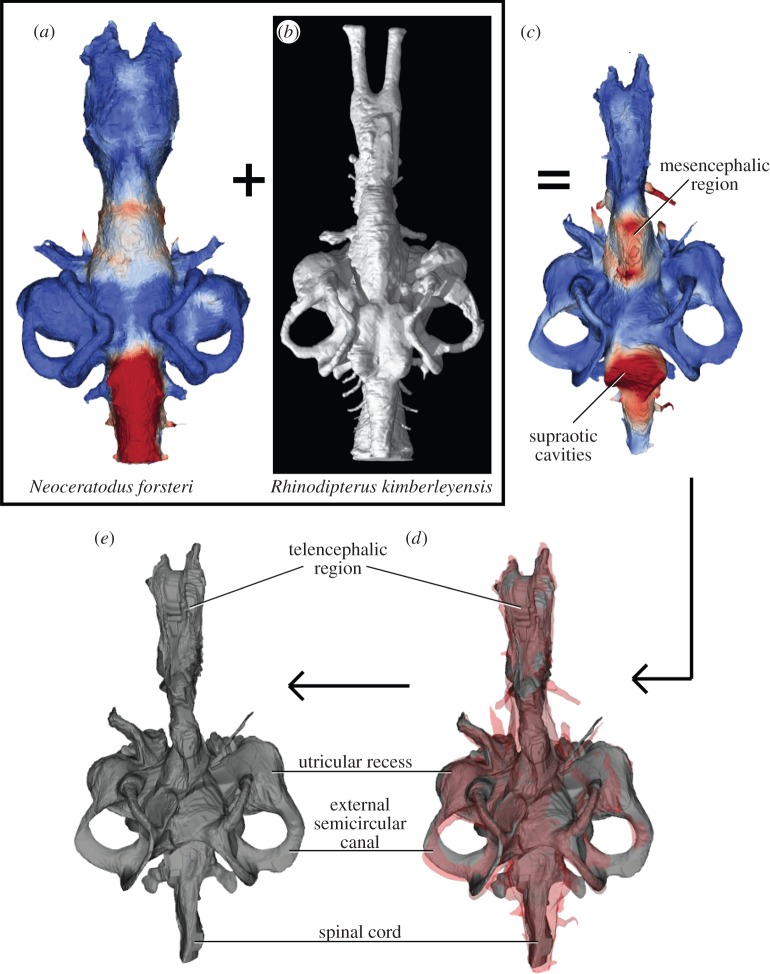
Endocast registration and warping analysis. (*a*) Colour-coded distance map for the relationship between brain and cranial cavity wall in the extant lungfish, *Neoceratodus* (adapted from [35]); (*b*) three-dimensional cranial endocast rendering of the Devonian lungfish, *Rhinodipterus* (adapted from [28]); (*c*) the reconstructed brain of *Rhinodipterus* presented as a colour-coded brain–endocast distance map; (*d*) spatial overlap of the reconstructed *Rhinodipterus* brain (grey) and endocast (pale red) and (*e*) reconstructed brain of the Devonian lungfish *Rhinodipterus* in dorsal view.

The three-dimensionally preserved cranium of *Rhinodipterus kimberleyensis* (figure 2*b*), Clement 2012 (WAM 09.6.149) came from the Late Devonian (Frasnian) Gogo Formation in north Western Australia [8]. The skull measures 52 mm in length. Dr Anne Warren discovered the specimen during the 2008 joint Museum Victoria/Australian National University (ANU) Gogo expedition led by Prof. John Long (funded by ARC Discovery Grant DP 0772138). Prof. John Long also prepared the specimen using baths of weak acetic acid to dissolve the surrounding limestone matrix, and strengthened newly exposed bones with Paraloid glue. The cranial endocast of *Rhinodipterus* was later described from three-dimensional tomographic scan data [28] and contributes half the material for the current study.

A formalin-fixed specimen of a juvenile Australian lungfish, *Neoceratodus forsteri* (ANU 73578) was obtained from Prof. Jean Joss of the former Lungfish Facility at Macquarie University, Sydney. The small cranium (measuring 9 mm in length) was immersed in 2% ethanolic iodine as a contrasting agent for six weeks in order to enhance differential soft tissue contrast following the methods of Metscher [36]. Both the brain and cranial endocast morphology of *Neoceratodus*, in addition to the spatial relationship between the two (figure 2*a*), was previously described and quantified [35]; this material comprises the second half of the materials used in our present investigation.

### 2.2. Tomographic methods

Both specimens were scanned at the ANU High-Resolution X-ray Computed Tomography facility [37] using the X-Tek RTR-UF225 X-ray source and Newport RV120PP rotation stage. A Roper PI-SCX100:2048 X-ray camera was used to record the radiographs. The *Rhinodipterus* skull was scanned with a spatial scan resolution of 55.5 µm, the iodine-treated *Neoceratodus* with a resolution of 16.5 µm. Three-dimensional segmentation and rendering of the *Neoceratodus* brain and both cranial endocasts were performed using the software VGStudio Max, v. 2.2 (Volume Graphics Inc., Germany), and the resulting three-dimensional volumes were later exported in STereoLithography format.

### 2.3. Endocast registration and warping

The brain–endocast relationship in *Neoceratodus* was warped so as to fit in the *Rhinodipterus* endocast using an image registration based on optimization of a parametric deformation field using Elastix [38]. The B-spline parameters were optimized using a stochastic gradient descent method with image intensities and 12 landmarks, corresponding points identified in both the *Rhinodipterus* and *Neoceratodus* endocast STereoLithographs (STLs), as input. The landmarks are given in the electronic supplementary material, figure S1 and table S2. A weighted sum of pointwise mean square intensity differences of segmented volume images of the *Rhinodipterus* and *Neoceratodus* endocasts and the mean square distance between the landmarks was minimized in the optimization process.

### 2.4. Presentation of data

Spatial overlap and surface distance between the endocast and the resulting reconstructed *Rhinodipterus* ‘brain’ were analysed using our previously presented software tool and using the same methodology of Clement *et al*. [35]. The distance map shows the distance between corresponding points on the superimposed models, and uses a divergent and perceptually linear colour map [39] to display larger distances in a warmer colour (see the electronic supplementary material, movie S3). Further quantitative analysis of the brain–endocast relationship was performed by computing the mean absolute surface distance, the maximum absolute surface distance [40], and the volume overlap (Dice similarity coefficient) [41].

## 3. Results

**Figure 3.**
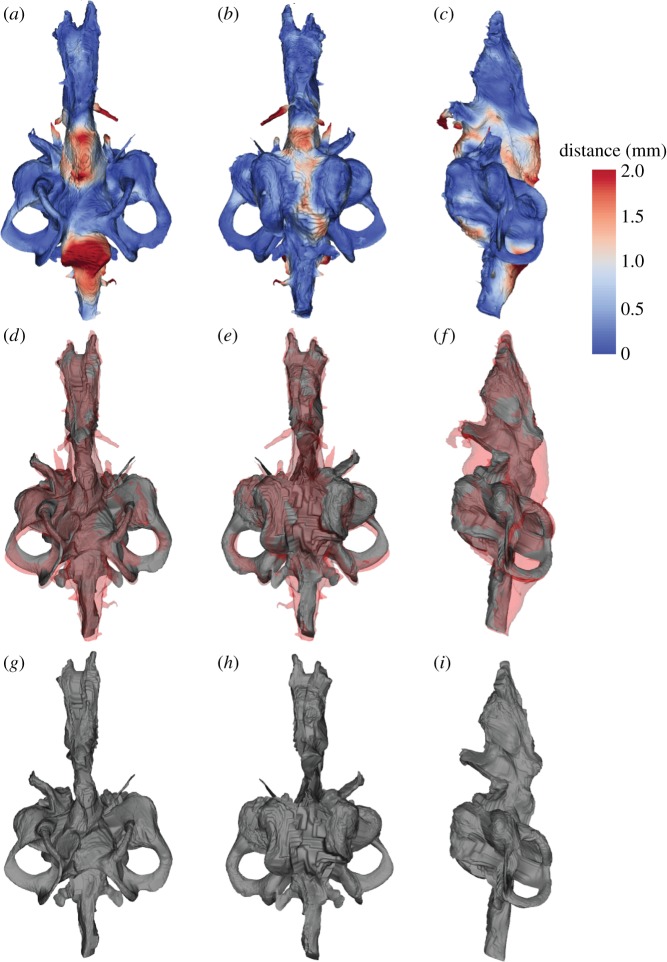
Brain morphology in *Rhinodipterus* as inferred from extant lungfish. The reconstructed brain of *Rhinodipterus* presented as a colour-coded brain–endocast distance map in (*a*) dorsal, (*b*) ventral and (*c*) lateral view. Spatial overlap of the reconstructed *Rhinodipterus* brain (grey) and endocast (pale red) in (*d*) dorsal, (*e*) ventral and (*f*) lateral view. Reconstructed brain of the Devonian lungfish *Rhinodipterus* in (*g*) dorsal, (*h*) ventral and (*i*) lateral view. Anterior to the top of page.

Figure 3 shows our reconstruction of the brain in the Devonian lungfish *Rhinodipterus*. Not surprisingly, it is reminiscent of its endocast in overall form; the forebrain region is elongate and narrow, comprising ≈50% of the total length, the midbrain is short (≈10%), while the hindbrain is relatively broad but also quite long (≈40%). The labyrinth region is the tallest brain region, and just slightly shorter than the hindbrain.

As the forebrain in *Neoceratodus* had such a close fit to the cranial endocast, the brain of *Rhinodipterus* is reconstructed with the same closeness of fit and therefore shows the distinct ventral bulge of the telencephalon and prominent hypophysis ventrally. As the anterior extent of the endocast of *Rhinodipterus* was not preserved, the nasal capsules have not been reconstructed here.

The mesencephalon in *Neoceratodus* was much smaller than the portion of the cranial cavity housing it, and is consequently the smallest brain region. Our reconstruction of *Rhinodipterus* here allows the same proportion of space between the midbrain and the cranial walls around it. There could not have been space for large optic lobes like those seen in actinopterygian endocasts [18–20].

The hindbrain in *Neoceratodus* was closely associated with the cranial cavity, particularly in the region between the two crus communes, but also along its ventral margin. We were able to match the position of the canals for the trigeminal and vagus nerves between the two datasets. There is a large space between the myelencephalon and the cranial cavity dorsally.

The labyrinth region in the *Rhinodipterus* endocast was well preserved, and this region showed close association in *Neoceratodus* so the inner ear can be reconstructed with relative certainty. *Rhinodipterus* is reconstructed as having had a large differentiated utriculus, common sacculolagenar pouch and three robust semicircular canals whose crus commune rises above the hindbrain roof. The sacculolagenar pouch in our reconstruction is somewhat anteriorly displaced and suggests a further registration point along its posterior extent should be included in any further analyses to increase reconstruction accuracy.

## 4. Discussion and conclusion

Overall, the quality of the brain reconstruction produced by our analysis is fairly satisfactory (figure 3). A difficulty when developing the image registration method includes the trade-off between matching image intensity and anatomic landmarks, and the degree of elasticity in the registration process. Higher elasticity gives better overlap between the resulting volumes, but at the cost allowing unnatural deformations. The model suffers from some asymmetries, notably affecting the dorsal surface between the inner ears, but these artefacts could probably be avoided by using a greater number of registration points. A more serious question is whether the model accurately reflects the shape of the brain of *Rhinodipterus*. Ultimately, this is of course unknown, pending the discovery of a preserved brain in this taxon (cf. [2,3]), but it is possible to examine the methodological validity of the reconstruction process.

In this specific instance, *Neoceratodus* is one of three living lungfishes (the other two being *Protopterus* and *Lepidosiren*) that together constitute the living sister group of *Rhinodipterus*. *Neoceratodus* is the most morphologically conservative of these lungfishes and its endocast does not differ greatly from that of *Rhinodipterus* in shape, suggesting that it is a well-chosen template for the reconstruction of *Rhinodipterus*. The only major cause for concern is the possible effect of allometric growth: the scanned *Neoceratodus* specimen is a very small individual, whereas the *Rhinodipterus* is large (and presumably adult), and it is possible that the size relationship between brain and cranial cavity would change from juvenile to adult in both genera [42]. However, comparison between the brain morphology of our juvenile *Neoceratodus* specimen with those of adults [43,44] we mostly find differences concerning length of the olfactory tracts and the shape of the telencephalon (for more details, see [35]), while the midbrain and hindbrain regions appear to remain of similar proportions throughout ontogeny. Indeed, we are well aware that brains scale allometrically (negatively) with body size. We must stress that far from reconstructing the brain morphology of *Rhinodipterus* flawlessly, this paper instead presents a methodology with which to reconstruct the gross brain morphology in any fossil representative using information from the closest extant species. With respect to lungfish, much further work is required to quantify ontogenetic changes in the extant taxa [45], and establish how we can interpret variability in brain morphology (whether this be due to evolutionary history, ontogeny, individual variation or behavioural parameters) when applying this methodology to fossil species.

In addition to a greater understanding of changes throughout ontogeny, it could be argued that a phylogenetically constrained approach, where templates of brain–endocast fit are generated for phylogenetic nodes and used to guide the reconstruction process of fossil taxa branching off the intervening internodes, would be more rigorous and would bring the process into line with current thinking on ancestral character state reconstruction [46]. In the present instance, this would involve reconstructing an ancestral template for the lungfish crown-group node (based on data from *Neoceratodus*, *Protopterus* and *Lepidosiren*), another for the lungfish total group node (based on templates for the lungfish crown group and tetrapod crown group), and then deriving a specific template for *Rhinodipterus* by some form of averaging the crown-group-node and total-group-node templates. As can easily be seen, this would require a substantial number of additional scanned endocasts from lungfishes, amphibians and amniotes, and can scarcely be recommended as a realistic approach at the present time. However, with the ever-increasing availability of tomographic datasets we predict that such comparative analyses will become a more practical and useful tool in the future.

## Supplementary Material

S1 Fig. Figure showing corresponding endocast landmark points used for registration. A, extant Australian lungfish, Neoceratodus, endocast in dorsal view; B, Late Devonian fossil, Rhinodipterus, endocast in dorsal view. Anterior is to the right.

## Supplementary Material

S2 Table. Table of landmarks used for endocast registration between Neoceratodus to Rhinodipterus. The endocast STL’s were converted into stacks of TIFF slices, from which corresponding landmarks are given as (x,y,z) coordinates from both the exterior surfaces of both endocasts.

## Data accessibility

All relevant data are available from the Dryad Digital Repository http://dx.doi.org/10.5061/dryad.820j2.
