# Supplementary material for: A new method for reconstructing brain morphology: applying the brain-neurocranial spatial relationship in an extant lungfish to a fossil endocast
Source: R Soc Open Sci. 2016 Jul 20;3(7):160307. doi: 10.1098/rsos.160307 (PMC4968476; doi:10.1098/rsos.160307)
Supplement: S2 Table. Table of landmarks used for endocast registration between Neoceratodus to Rhinodipterus. The endocast STL’s were converted into stacks of TIFF slices, from which corresponding landmarks are given as (x,y,z) coordinates from both the exterior surfaces of both endocasts. [file rsos160307supp2.docx]

**Supplementary Information 2: Table of landmarks used for endocast registration between *Neoceratodus* to *Rhinodipterus*.**

| **No.** | **Landmark** |  | ***Neoceratodus*** | **ANU 73578** | ***Rhinodipterus*** | **WAM 09.6.149** |
| --- | --- | --- | --- | --- | --- | --- |
|  | **Description** | **Side** | **Slice no.** | **Co-ordinates** | **Slice no.** | **Co-ordinates** |
| 1 | Junction between olfactory lobes | - | 340/373 | 58, 73, 339 | 128/682 | 160, 117, 127 |
| 2 | Base of optic nerve (II) | Left | 256/373 | 86, 110, 255 | 202/682 | 188, 223, 201 |
| 3 | Base of optic nerve (II) | Right | 258/373 | 75, 46, 257 | 245/682 | 173, 195, 244 |
| 4 | Ventral extent of hypophysial fossa* | - | 195/373 | 109, 78, 194 | 284/682 | 251, 221, 283 |
| 5 | Base of Trigeminal nerve (V) | Left | 178/373 | 83, 119, 177 | 277/682 | 158, 325, 276 |
| 6 | Base of Trigeminal nerve (V) | Right | 168/373 | 62, 62, 167 | 346/682 | 130, 281, 345 |
| 7 | Lateral extent of utriculus | Left | 139/373 | 91, 189, 138 | 305/682 | 184, 492, 304 |
| 8 | Lateral extent of utriculus | Right | 130/373 | 50, 1, 129 | 439/682 | 100, 226, 438 |
| 9 | Lateral extent of horizontal scc | Left | 109/373 | 67, 195, 108 | 371/682 | 174, 575, 370 |
| 10 | Lateral extent of horizontal scc | Right | 102/373 | 21, 17, 101 | 526/682 | 58, 283, 525 |
| 11 | Base of Vagus nerve (X) | Left | 63/373 | 58, 134, 62 | 463/682 | 147, 502, 462 |
| 12 | Base of Vagus nerve (X) | Right | 57/373 | 42, 83, 56 | 519/682 | 107, 458, 518 |

The endocast STL’s were converted into stacks of TIFF slices, from which corresponding landmarks are given as (x,y,z) coordinates from both the exterior surfaces of both endocasts. *indicates point not shown in SI2 (figure)
